# The stuck haemodialysis catheter—a case report of a rare but dreaded complication following kidney transplantation

**DOI:** 10.1186/s12882-024-03507-z

**Published:** 2024-03-18

**Authors:** Cameron Burnett, S. Chandler, D. Jegatheesan, B. Pearch, A. Viecelli, D. W. Mudge

**Affiliations:** 1 Department of Kidney and Transplantation Services, Princess Alexandra Hospital, Woolloongabba, QLD Australia; 2 Department of Interventional Radiology, Princess Alexandra Hospital, Woolloongabba, QLD Australia; 3 PA-Southside Clinic Unit, Faculty of Medicine, The University of Queensland, Brisbane, Australia

**Keywords:** Central cuffed catheter, Haemodialysis, Stuck permcath, Interventional radiology, Case report

## Abstract

**Background:**

Tunnelled cuffed haemodialysis catheters are at increased risk of incarceration or becoming ‘stuck’ via fibrotic adhesion to the central veins when left in situ for prolonged periods of time. Stuck catheters cannot be removed using standard techniques such as bedside dissection of the cuff. Whilst there are several strategies published for the removal of these incarcerated lines, there is no consensus on the best approach. Here we present a challenging case of a stuck haemodialysis catheter in the acute post transplantation period.

**Case Presentation:**

A 66-year-old female on haemodialysis presented for kidney transplantation with a tunnelled-cuffed haemodialysis catheter in situ for five years. Following transplantation, removal of the line was unsuccessful despite dissection of the cuff, with traction causing a choking sensation with tracheal movement. Eventually, the line was removed without complications utilising sequential balloon dilatation by interventional radiology and the patient was discharged without complications.

**Conclusions:**

This case serves as a timely reminder of the risks of long-term tunnelled haemodialysis catheters and as a caution towards proceeding with kidney transplantation in those with long-term haemodialysis catheters in situ. Greater nephrologist awareness of interventional radiology techniques for this challenging situation will help to avoid more invasive strategies. The risks of a stuck catheter should be included in the discussions about the optimal vascular access and transplantation suitability for a given patient.

## Background

Tunnelled central venous dialysis catheters are typically considered a temporary vascular access choice for dialysis patients awaiting definitive dialysis access such as an arteriovenous fistula (AVF) for haemodialysis or a peritoneal dialysis catheter. However, there are certain circumstances where tunnelled catheters are considered necessary after exhaustion of other access options, as outlined in the KDOQI 2019 vascular access guidelines [1]. The removal of tunnelled haemodialysis catheters is typically a straightforward procedure with either removal with gentle traction or dissection of the cuff performed under local anaesthesia at the bedside. However, tunnelled venous catheters that remain in situ long-term can result in the development of fibrotic adhesions between the line and the superior vena cava or right atrial free wall which can make removal difficult or impossible.

The incarcerated, hereafter referred to as ‘stuck’, tunnelled-cuffed venous catheter is an uncommon but challenging situation, where the line cannot be removed successfully using standard techniques. Stuck catheters are reported as a complication in around 1% of haemodialysis catheters, but have been reported as high as 20% in lines in situ for over 2 years [2, 3]. Attempting removal using excessive force has previously resulted in vessel perforation, right atrial tears, hemopericardium and retained fragments [4, 5]. Here we present a case of an incarcerated haemodialysis catheter in the acute post transplantation period and discuss the role of long-term catheterisation and the implications for patient care.

## Case presentation

### History and initial presentation

The patient was a 66-year-old female on maintenance haemodialysis with a history of lupus nephritis, hypertension and post-menopausal osteoporosis. She had no previous lines or central vascular devices. She had been maintained on haemodialysis via a left internal jugular tunnelled cuffed catheter that had been in situ for five years due to patient preference. She was clinically well when she presented for deceased donor kidney transplantation.

### Hospital course, follow-up and outcome

**Fig. 1:**
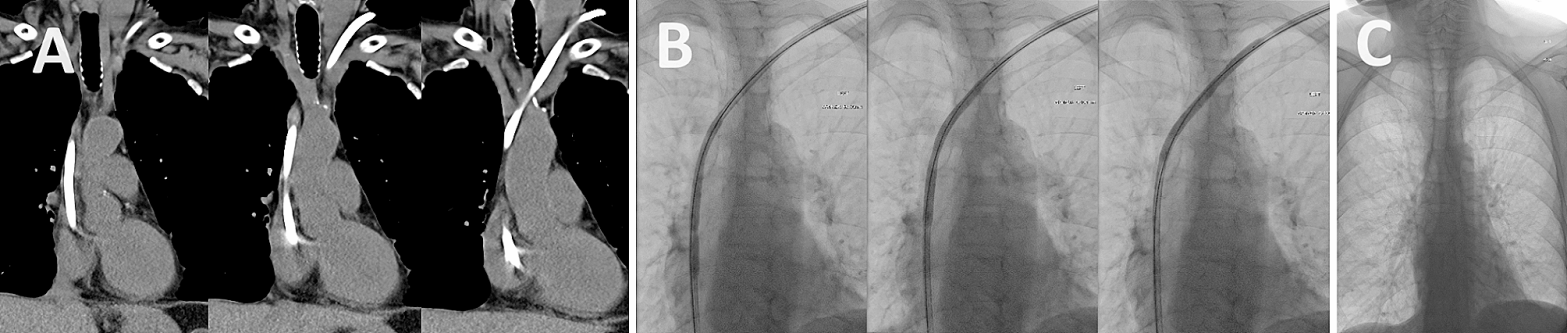
Peri-operative imaging of the incarcerated dialysis catheter. (A) CT pre-assessment, (B) Fluroscopic balloon dilation, (C) Post-operative chest x-ray

The patient underwent kidney transplantation with immediate graft function and without medical or surgical complications. On day 5 postoperatively, the line was planned for routine removal, and attempted at the bedside with local anaesthesia infiltration. Following uncomplicated dissection of the cuff, tension on the catheter resulted in a choking sensation of the patient together with evident ipsilateral movement of the midline structures including the trachea. Further attempts at the bedside, and later in theatre by vascular and cardiothoracic surgeons had the same result. A CT venogram was performed demonstrating a contracted superior vena cava around the vascular catheter as it passed into the right atrium (Fig. 1A). An initial attempt by interventional radiology with dissection of the tract using blunt forceps and fluoroscopy was abandoned after fluoroscopy again revealed tracheal displacement and discomfort with attempted traction. Laser sheath removal was considered but was unsuitable due to the excessive size of the patient’s haemodialysis catheter (7 mm; maximal line diameter for available laser systems was 5 mm).

Following multidisciplinary team discussion between vascular, cardiothoracic and interventional radiology teams, a decision was made to undertake a second fluoroscopic technique using the *Hong technique* [7]. A guidewire was passed down into the IVC from a lumen of the central venous catheter and a 6 mm balloon dilatation was undertaken sequentially from the distal to proximal component of the line (Fig. 1B). Following this, the line was able to be removed safely (Fig. 1C). The patient suffered no complications and was discharged home on day 8 post transplantation.

Coronal CT scan imaging demonstrating the course of the haemodialysis catheter entering the left internal jugular vein posterior to the sternocleidomastoid, through the brachiocephalic vein and through a contracted superior vena cava with suspected fibrotic tethering as the likely cause of incarceration (A). Fluoroscopic IR images of 6 mm sequential balloon dilation via an intraluminal guidewire (Hong Technique), intending to disrupt fibrotic tethering and treat central venous stenosis (B). Post-operative chest x-ray demonstrating successful removal of the line without complications (C).

## Conclusions

Stuck catheters are becoming increasingly common due to the widespread use of tunneled catheters. Stuck catheters are typically diagnosed by nephrologists and often prompt multispecialty involvement, usually from vascular or cardiothoracic surgeons or interventional radiology (IR). Stuck catheters are understood to become tethered, typically to the superior vena cava, by fibrotic adhesions and stenosis that develop secondary to neointimal hyperplasia from shear forces relating to turbulent blood flow [3]. This is congruent with the situation that occurred in our patient and was demonstrated on our CT venogram outlined in Fig. 1. The first successful IR approach for the removal of stuck haemodialysis catheters was published in 2011 and was named the *Hong technique*. A guidewire is first introduced through a lumen of the affected line, followed by sequential endoluminal balloon dilatation along the length of the catheter, presumably disrupting fibrotic bands and concurrently treating central stenosis allowing easier removal of the line [5, 7]. Several case reports have since documented the use of modified Hong techniques or other balloon and snare strategies for stuck central lines of different kinds with excellent outcomes [3, 5, 7, 8, 9]. This include d a timely and uncomplicated removal of a stuck hemodialysis catheter tethered amidst pacemaker leads [8], suggesting IR as a favourable first-line approach.

Historically, rescue approaches for stuck catheters included “cutting and burying” the catheter to leave a portion in situ, or surgical removal following sternotomy [6]. There are limited data available on the safety and long-term outcomes of thoracotomy for stuck haemodialysis catheters. A recently published survey [2] of 72 stuck haemodialysis catheters across 30 centres in Italy revealed that 11 patients were not subject to advanced procedures, with 3 lines left in situ and the other 8 were buried. There was a 77% success rate for stuck catheters undergoing advanced procedures (47 were removed and 14 were left in situ or buried). Success rates were slightly higher (87%) in those undertaking sequential balloon dilation specifically (26 of 30 lines). However, 4 patients (13%) whom this approach was used had complications including a haemopericardium, catheter fragmentation, guidewire entrapment and severe pain requiring advanced anaesthetic support, suggesting that utilization of advanced procedures for stuck catheters should be done at a centre with advanced resuscitation support with access to intensive care and cardiothoracic surgery. Over 70% of the stuck catheters were at least 24 months old, suggesting *time in situ* is a risk factor for line incarceration. In terms of the feasibility and safety of burying these catheters, a retrospective case series examined the safety of cutting and burying stuck catheters in six patients, with mixed outcomes. Three of these patients needed anticoagulation for a chronic organising thrombus adjacent to the retained line and developed central stenosis, two of these patients successfully underwent transplantation with retained lines in situ. Of the three remaining patients, two eventually had the line removed by cardiothoracic surgery without the need for thoracotomy, and the last patient died of line-related sepsis. This approach predisposes to ongoing line-related access complications and should be considered as a last resort, although further observational data would provide insight into the longer-term safety of this approach.

Retrospective analyses have demonstrated that patients with stuck haemodialysis catheters often proceed directly to sternotomy or internalization of a retained line without being offered an IR approach first. This is probably a reflection of variability in training and experience with managing stuck haemodialysis catheters as well as clinical characteristics of the patients [6]. *Forneris and colleagues* have provided a thorough overview of safe management for stuck haemodialysis catheters, suggesting that unfamiliarity of IR techniques or limited experience in stuck dialysis catheters may contribute to unnecessary internalization or sternotomy [10, 11]. In centers limited by geographic considerations or resources, transfer to an IR-capable facility should be considered along with the patient characteristics prior to internalization. Among the options considered for removal of a stuck catheter, endoluminal sequential balloon dilatation appears to be the best approach [2]. Nephrologists should be aware of the utility of such techniques and consider its feasibility prior to discussing the best management strategy with the patient.

The KDOQI 2019 vascular access guidelines suggest that dialysis catheter utilization rates be limited to less than 20% of kidney failure patients, yet some centers have published prevalence as high as 42.9%, with a third of patient refusing AVF creation or subsequent revision or replacement [12, 13]. In a survey of perceived barriers and attitudes toward AVF creation and use, reasons for refusal varied from concern about the risks of surgery (42.5%), lack of understanding (23.3%), fear of needles (15.1%), denial of disease or need for HD (17.8%) and cosmetic reasons (1.4%) [13]. Naturally, prevention of long-term tunneled haemodialysis catheter use would reduce stuck catheter incidence, while poor fistula maturation, vessel quality and patient refusal are risk factors for dialysis catheter use long term [12, 13]. Improved nephrologist awareness to this complication is important in prioritizing and facilitating arteriovenous access creation, including an exploration and addressment of potentially modifiable reasons for patients that refuse fistula creation. The risks of a stuck haemodialysis catheter should be included in discussion when considering the optimal vascular access for a given patient, whilst also considering individual characteristics and goals of care.

Careful consideration should be made when assessing these patients for transplantation suitability considering the risks associated with stuck dialysis catheters. Fortunately, our patient tolerated the procedure well without complication. However, attempting to remove stuck dialysis catheters acutely following transplantation lends itself to unique considerations. Complications arising from advanced procedures including fragmentation or hemopericardium may place the patient at significant risk of morbidity including infection as well as chronic organizing thrombosis and potentially mortality. Further procedures may worsen graft dysfunction secondary to hypoperfusion or precipitate the need for blood transfusions. This may in turn increase sensitization, increasing the risk of rejection and complicating future transplantation. Furthermore, manipulation of the tract and introducing guide wires through a central line peri-operatively may carry a higher infection risk post immunosuppression and extended intravenous antibiotic coverage could be considered for difficult cases.

## Data Availability

No datasets were generated or analysed during the current study.
